# Transition in the Cause of Fever from Malaria to Dengue, Northwestern Ecuador, 1990–2011

**DOI:** 10.3201/eid1910.130137

**Published:** 2013-10

**Authors:** Sara G. Cifuentes, James Trostle, Gabriel Trueba, Meghan Milbrath, Manuel E. Baldeón, Josefina Coloma, Joseph N.S. Eisenberg

**Affiliations:** Universidad San Francisco de Quito, Quito, Ecuador (S.G. Cifuentes, G. Trueba, M.E. Baldeón);; Trinity College, Hartford, Connecticut, USA (J. Trostle); University of Michigan, Ann Arbor, Michigan, USA (M. Milbrath, J.N.S. Eisenberg);; University of California Berkeley, Berkeley, California, USA (J. Coloma)

**Keywords:** dengue virus, dengue fever, malaria, etiology, reemergence, ELISA, reverse transcription PCR, surveillance, Ecuador, febrile, viruses, vector-borne infections

## Abstract

In tropical areas, the predominant cause of fever has historically been malaria. However by 2011, among febrile patients in northwestern Ecuador, dengue was identified in 42% and malaria in none. This finding suggests a transition in the cause of fever from malaria to other illnesses, such as dengue.

Clinical and public health decisions about infectious diseases depend on the specific agents of infection. The predominant cause of fever in tropical areas has historically been malaria. However, dengue is becoming more of a concern as its geographic range expands, despite efforts to control the spread of the main vector, *Aedes aegypti* mosquitos (*1*). In the past 2 decades, dengue has expanded from urban areas, the focal point of endemic and epidemic activity, to more rural regions (*2*). At about the same time, malaria incidence has decreased by 17% globally; in Ecuador, it decreased by >75% (*3*). Etiologic transitions like these require timely changes in treatment and intervention strategies. 

In Ecuador, reported dengue cases reemerged in 1988, peaked in 2000, and exhibited typical oscillatory patterns during the next 12 years (Figure 1). During the 1990s, dengue spread to the province of Esmeraldas; after 1998, when *Ae. aegypti* mosquitoes became established, dengue spread into the rural Esmeraldan community of Borbón (*4*). According to official data, the number of confirmed dengue cases in Borbón remained low until 2009 (*5*). Although reporting bias probably affects these data, the reported pattern is consistent with the national epidemic profile for 2009–2010 (Figure 1). During this same period, malaria prevalence decreased steadily in Esmeraldas and throughout Ecuador (Figure 2).

**Figure 1:**
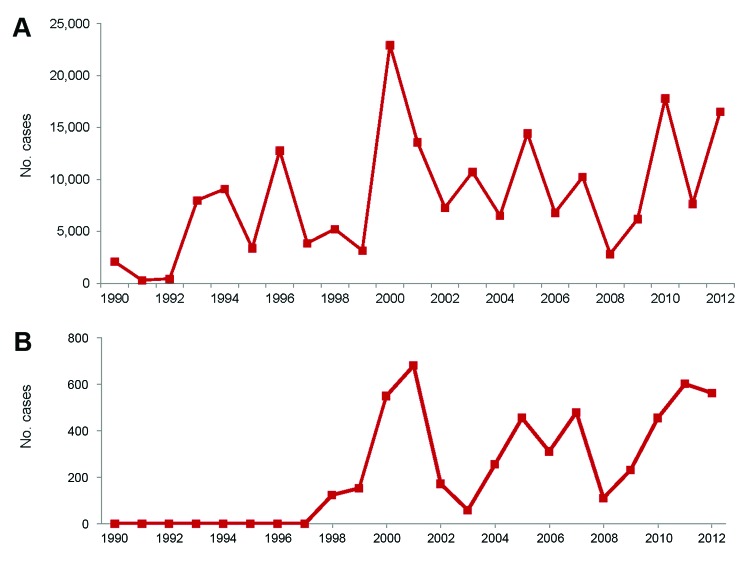
Suspected cases of dengue in Ecuador (A) and Esmeraldas Province (B), 1990–2012. Data from Annual Epidemiology Reports, Ministerio de Salud Pública del Ecuador.

**Figure 2:**
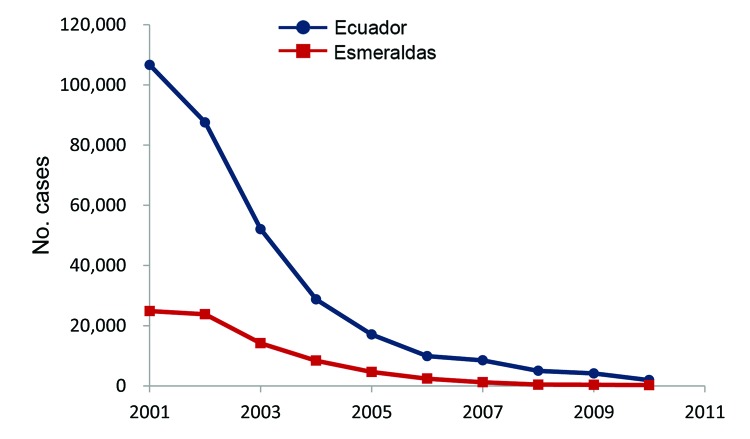
Confirmed malaria cases in all of Ecuador and in the Esmeraldas Province, 2000–2010. Data from the Department of Epidemiology, Servicio Nacional de Erradicaciòn de la Malaria, Ministerio de Salud Pública del Ecuador.

In Ecuador, since the 1988 reintroduction of dengue virus, all 4 serotypes have circulated. In contrast, dengue virus type 1 (DENV-1) was introduced into Esmeraldas in 1990 and was the only documented serotype circulating until 2004, when DENV-4 was identified. DENV-4 was thought to arrive in Esmeraldas from Colombia and subsequently spread into the rest of Ecuador (*6*). The most likely reason Esmeraldas was a point of DENV introduction is the increased building of roads during 1996–2002; these roads connected Esmeraldas to Colombia and the rest of Ecuador and are a major reason for pathogen transmission (*7*,*8*).

Most surveillance data are predicated on clinical manifestations. Like many other infectious diseases, dengue infection follows an unpredictable clinical evolution, ranging from inapparent or mild fever to severe or fatal hemorrhagic disease (*9*). Patients with acute dengue infection experience broad clinical signs and symptoms (e.g., fever, headache, myalgia, and abdominal pain), often characterized as an acute undifferentiated febrile illness, which can be caused by a wide array of pathogens, including *Leptospira* spp., *Plasmodium* spp., *Rickettsia* spp., and DENV (*10*); therefore, without laboratory tests, dengue infections are often misdiagnosed (*11*).

Given the recent downward trend in the number of malaria cases and the limited official data available on confirmed dengue cases in rural Esmeraldas, our objective was to determine whether dengue infection is present in febrile patients at either the laboratory of the Servicio Nacional de Erradicación de Vectores Artrópodos (SNEM), where patients who suspect that they have malaria often go, or the Hospital Civil de Borbón (HCB), the main hospital in the region. We provide data from Borbón, a rural community in northwestern coastal Ecuador, as a case study of this etiologic transition of fever from malaria to other illnesses, such as dengue. In addition, we provide serotype-specific data that describe the directional flow of dengue at the regional and national levels.

## The Study

At HCB, as part of the routine workup for febrile patients, serum samples were collected, stored at −20°C, and transported in liquid nitrogen to Quito for PCR. Hospital personnel also tested these samples by using a dengue IgM/IgG-capture ELISA with high specificity and sensitivity (Panbio, Waltham, MA, USA). At the SNEM laboratory, 2–4 drops of whole blood were collected on filter paper (Whatman 903, Kent, UK) from persons spontaneously seeking or referred for malaria diagnosis. Filter papers were left to dry overnight, stored at 0°C in a zipper bag, and transported to Quito for analysis. DENV was amplified from total RNA from blood spots and serum as described (*12*,*13*). Enriched media from DENV 1–4 cultures were used as positive controls. Quality of RNA extracts was tested by using β-actin gene amplification. PCR products were sized in 1.5% agarose gel electrophoresis and SYBR Safe DNA Gel Stain (Invitrogen, Carlsbad, CA, USA) (1:10,000) under UV light. PCR product was sent to Functional Biosciences (Madison, WI, USA) for sequencing. All participants provided oral consent. The study was approved by institutional review board committees at the Universidad San Francisco de Quito (Quito, Ecuador) and the University of Michigan (Ann Arbor, MI, USA).

From July 2010 through February 2011, a total of 77 samples (36 serum, 41 blood spot) were collected from febrile patients. Of these 36 hospital patients, 10 were from Borbón and 26 were from the surrounding communities; patients were 2–74 years of age (median 19 years), and fever duration was 3–20 days (median 6 days). Six (17%) of the 36 serum samples and 7 (17%) of the 41 blood spots were positive for dengue by PCR; whereas 10 (29%) of the 34 serum samples tested by ELISA were positive for dengue IgM (Table). The ages of the 10 patients with positive results by ELISA were 3–63 years (median 38 years), and fever duration was 4–13 days (median 6.5 days). Although positive PCRs are sufficient indicators of acute infection, the positive IgM reflects a cumulative incidence over 60–90 days. Therefore, the 17% PCR-positive estimate is a lower bound, and the 42% estimate by PCR or ELISA is an upper bound. The DENVs in the 13 samples that were positive by PCR were either serotype 2 or 3; samples from 1 patient contained both serotypes, probably resulting from a co-infection. Each institution (HCB and SNEM) submitted samples from the same 2 patients, but only 1 was positive for dengue by PCR. During this same period, DENV-1, -2, and -4 were detected in coastal cities in other regions of Ecuador (DENV-3 has not been reported in Ecuador since 2009 [*14*]). These data suggest that DENV-3 was introduced into the study region from another country. Colombia is a likely source, given the proximity of the study region to Colombia and the fact that DENV-3 was circulating in Colombia; but without sequence data, the geographic location of the source cannot be confirmed.

**Table:** Dengue virus in serum samples and blood spot samples, Ecuador

**Specimen**	**No. specimens**		**No. positive **			**No. each dengue virus subtype**			
			ELISA	Reverse transcription PCR		1	2	3	4
**Serum**	36		10*	6†		0	5	1	0
**Blood spot**	41		Not applicable	7‡		0	3	5	0

*Only 34 serum samples were tested** by ELISA; only 1 of the 10 positive samples was also positive by PCR.** **†Five samples were from male patients.** ‡Three samples were from male patients. One patient was co-infected with both serotypes.

## Conclusion

This study provides evidence of a transition of febrile disease etiology from *Plasmodium* spp., traditionally highly prevalent in this area, to reemerging pathogens, such as DENV. Other possible fever-causing agents for which we did not test include *Leptospira* spp.*, Rickettsia* spp., *Brucella* spp., and viral agents of encephalitis (*11*,*15*). Given that malaria has historically been the predominant cause of fever in the developing world, febrile patients are often triaged toward malaria treatment, especially in resource-poor areas. Moreover, the awareness of malaria is embedded culturally and behaviorally in these communities: having a fever is equated with having malaria. Of the 40 febrile patients who sought treatment for malaria, none had malaria but 17% had dengue (positive for DENV by PCR). Further research is needed to refine this estimate and explore how the Ministry of Health and the general population should be directed to manage cases of fever during this etiologic transition. The need to bring attention to this transition is exemplified by our finding of DENV-3 in our study site; this serotype has not been isolated in other parts of Ecuador since 2009. Esmeraldas, therefore, may be a major source of newly introduced dengue serotypes into Ecuador.
